# Gonorrhœa

**Published:** 1879-04

**Authors:** 


Gonorrhœa.—Bauer's method of treating gonorrhœa—based upon the theory that it is a purely local disease, the protecting layer of epithelium being thrown off, and the epithelial cells being converted into pus cells and discharged, leaving the mucous membrane exposed—is simply the use of a bland injection, which is followed by immediate relief to the pain, and usually results in a cure in about six days as follows:

R.—Inf. flax seed . . . ℥ vj.

Watery ext. opium . . . gtts. xviij.

M.—To be injected warm every three hours and retained for a few minutes.—*Western Lancet.*

Insomnia.—In cases of insomnia from anxiety, melancholia, or a low state of nutrition, camphor will act favorably when chloral, morphia, potassium bromide, etc., fail. One and a half to two or three grains may be subcutaneously injected after dissolving in almond oil.—*New York Med. Record.*

